# Comparative Oligo‐FISH Mapping Illuminates Chromosomal Evolution Among Rutaceae Species Diverged Over 50 Million Years

**DOI:** 10.1002/advs.202521629

**Published:** 2026-05-07

**Authors:** Li He, Hainan Zhao, Xiaoxue Zeng, Zujun Yang, Wenting Li, Guoyan Zhang, Guangrong Li, Jian He, Bin Guan, Jiming Jiang

**Affiliations:** ^1^ Horticulture Research Institute National‐local Joint Engineering Laboratory of Citrus Breeding Cultivation Key‐Laboratory of Horticultural Crops Biology and Germplasm Enhancement in Southwest China Ministry of Agriculture and Rural Affairs Key Laboratory for Germplasm Innovation & Utilization of Horticultural Crops of Sichuan Province Sichuan Academy of Agricultural Sciences Chengdu China; ^2^ State Key Laboratory of Maize Bio‐breeding Key Laboratory of Genome Editing Research and Application Ministry of Agriculture and Rural Affairs National Maize Improvement Center Frontiers Science Center for Molecular Design Breeding (MOE) College of Agronomy and Biotechnology China Agricultural University Beijing China; ^3^ Center for Informational Biology School of Life Science and Technology University of Electronic Science and Technology of China Chengdu China; ^4^ Department of Plant Biology Department of Horticulture Michigan State University AgBioResearch Michigan State University East Lansing Michigan USA

**Keywords:** chromosome evolution, chromosome painting, comparative karyotyping, molecular cytogenetics

## Abstract

Comparative fluorescence in situ hybridization (FISH) mapping, particularly chromosome painting, was first developed in mammalian systems and generated much of the foundational knowledge on chromosomal relationships among diverse mammalian species prior to the genome sequencing era. In plants, the advent of region‐ and chromosome‐specific FISH probes based on massively synthesized oligonucleotides (oligos) has greatly expanded the utility of FISH in cytogenetic mapping. We developed oligo‐based barcode‐FISH and chromosome painting probes in the model *citrus* species *Citrus maxima* and applied them to 13 species from the Aurantioideae subfamily of Rutaceae. All 13 species retained complete chromosomal synteny with *C. maxima* despite ∼20 million years of divergence. Remarkably, these probes were also successfully applied to *Boenninghausenia albiflora* (Rutoideae subfamily), which diverged from *citrus* species ∼52 million years ago. Comparative FISH mapping revealed the mechanism underlying its change in basic chromosome number from 9 to 10 and identified three distinct chromosomal translocation events in *B. albiflora*. Together, these results demonstrate that oligo‐based FISH probes developed in a model plant species can be effectively applied across deeply diverged lineages, enabling the rapid reconstruction of chromosomal evolutionary histories on timescales previously inaccessible to plant cytogenetics.

## Graphical abstract

Oligonucleotide‐based fluorescence in situ hybridization probes were developed in the model *citrus* species *Citrus maxima*. These probes were applied to comparative karyotyping across 14 species in the Rutaceae family. This analysis revealed chromosomal evolution in lineages that diverged from *Citrus* nearly 52 million years ago.

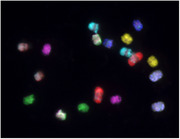

## 1 Introduction

Fluorescence in situ hybridization (FISH), developed more than 40 years ago [1], remains one of the most enduring and versatile techniques in genetics research. FISH using a DNA probe specific to an entire chromosome is commonly referred to as chromosome painting. Comparative chromosomal painting (CCP), also known as cross‐species chromosome painting or Zoo‐FISH, has been a transformative cytogenetic technique that was first developed in mammalian species [2, 3]. The method involves generating chromosome‐specific DNA probes, initially from flow‐sorted chromosomes of a “reference” species, labeling them with distinct fluorochromes, and hybridizing them to metaphase chromosomes of a “target” species. By visualizing conserved blocks of homology, CCP enables the identification of shared synteny, detection of chromosomal rearrangements, and reconstruction of karyotype evolution across diverse taxa [4, 5, 6].

The earliest CCP breakthroughs arose from applying human chromosome painting probes to the chromosomes of great apes (chimpanzee, gorilla, orangutan), which confirmed the high degree of synteny among hominids and famously demonstrated that human chromosome 2 originated from a head‐to‐head fusion of two ancestral ape chromosomes [3]. Soon after, the CCP between human and mouse produced the first high‐resolution comparative genome map between two distantly related mammals, revealing extensive reshuffling of chromosome segments since their divergence ∼80–100 million years ago (Mya) [7, 8]. Over the following decade, painting probes were developed for a wide range of mammals, including cat, dog, pig, cow, horse, and various marsupials, enabling direct comparisons of chromosomal evolution within and between orders [5, 9, 10, 11, 12]. These studies provided much of the foundational information on chromosomal relationships among mammalian species prior to the genome sequencing era [5, 6, 13].

FISH was introduced into plants more than 30 years ago [14, 15], but early applications were hampered by the lack of robust DNA probes [16]. This limitation was partially alleviated by the development of bacterial artificial chromosome (BAC) libraries, which provided chromosome‐anchored BAC clones as effective FISH probes [17, 18]. Initial attempts to develop chromosome painting probes in plants—using DNA from flow‐sorted chromosomes—were largely unsuccessful, primarily due to the high abundance of repetitive DNA sequences, especially in plant species with large complex genomes [16, 19]. A breakthrough came with the creation of painting probes in *Arabidopsis thaliana* by pooling large numbers of BAC clones specific to a single chromosome [20]. However, this strategy was largely restricted to species with small genomes and relatively low repeat content [21, 22, 23]. In large, repeat‐rich genomes, BAC pools contained excessive repetitive DNA, preventing their uses as chromosome painting probes.

The advent of oligonucleotide (oligo)‐based probes revolutionized the FISH techniques in plants [24]. Short oligos (40–60 nt) specific to chromosomal regions or entire chromosomes can be computationally identified and massively synthesized [25]. Consequently, versatile oligo‐based probes can now be designed for virtually any plant species with a sequenced genome. Two major classes of oligo‐based probes have emerged. The first, “barcode probes,” are derived from distinct regions across all chromosomes within a species [26]. Barcode probes yield unique signal patterns that enable unambiguous identification of all chromosomes in a species. Barcode‐FISH has been widely applied across diverse plant species for chromosome identification and karyotyping [27, 28, 29, 30, 31, 32, 33, 34, 35, 36, 37, 38]. The second class, “painting probes,” consists of oligos spanning entire chromosomes, and has been reported in an increasing number of plant species for diverse chromosomal research [39, 40, 41, 42, 43, 44, 45, 46, 47, 48, 49].

The Rutaceae family comprises 151 genera and more than 1600 species [50]. It includes several important fruit crops, such as pomelo (*Citrus maxima*), mandarin orange (*Citrus reticulata*), and orange (sweet orange, *Citrus sinensis*). A complete set of nine chromosome painting probes was developed in the model *citrus* species *C. maxima* (2n = 2x = 18) [43]. To expand this toolkit, we developed two barcode‐FISH probes in *C. maxima*, enabling the identification of all nine *citrus* chromosomes in a single FISH experiment. We applied these probes across a set of 14 distantly related species to investigate karyotype evolution in Rutaceae. Our analyses reveal strong karyotype conservation among 13 species in the subfamily Aurantioideae, which has a crown age of ∼19.8 million years (Ma) (12.1–28.2 Ma) [51]. We further investigate the karyotype of *Boenninghausenia albiflora*, a species from the subfamily Rutoideae that diverged from *citrus* more than 50 million years ago (Mya). Our results uncover the chromosomal rearrangements underlying the distinct karyotype of *B. albiflora* (2n = 2x = 20) compared to *C. maxima* (2n = 18). Collectively, these findings demonstrate that oligo‐based probes developed in a model plant species can be applied to far beyond closely related taxa, providing new opportunities to trace chromosome evolution across deep timescales in plants.

## 2 Results

### 2.1 Evolutionary Divergence of Rutaceae Species Selected for Comparative Karyotyping

**FIGURE 1:**
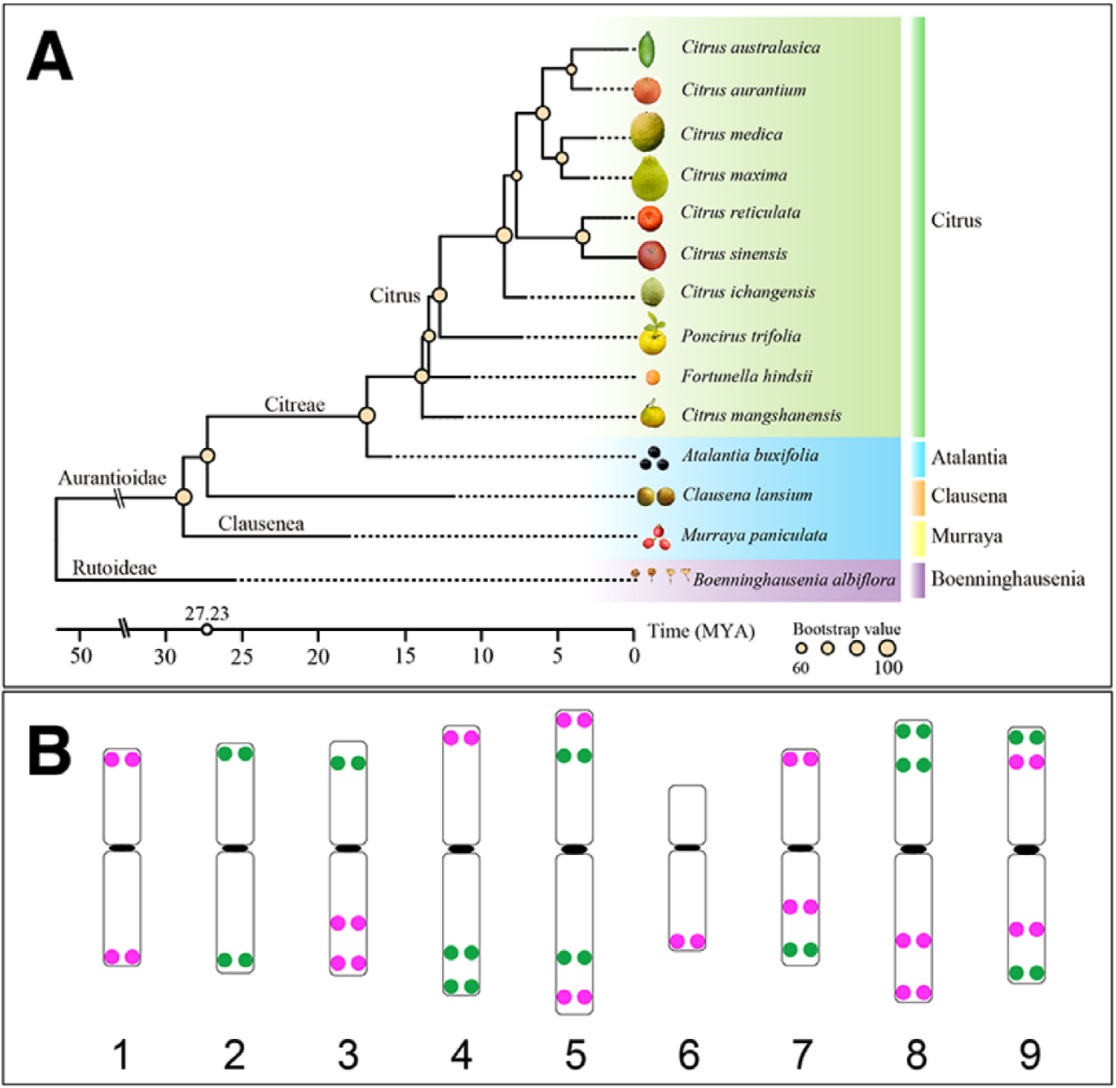
Plant species and barcode design for barcode‐FISH study. (A) Phylogenetic tree of 14 species from the subfamilies Aurantioidae and Rutoideae used in this study. The tree was constructed by MrBayes, consisting of one Rutoideae species (purple), three species from *Citrus*‐related genera (blue), and ten species from the *Citrus* genus (green). (B) Predicted barcode‐FISH signals on the nine *Citrus* chromosomes. The ideogram is based on physical lengths of the nine pseudomolecules of *C. maxima* and the position of the 26 barcode regions on the chromosomes (Table S1).

We previously established karyotypes for six different *Citrus* species using chromosome painting probes developed in *C. maxima*. These species retained complete chromosomal synteny despite being diverged more than 9 Mya [43]. To investigate chromosomal synteny over deeper evolutionary timescales, we selected seven additional, more distantly related species from the subfamily Aurantioideae. These species (Figure 1A) diverged approximately 20 Mya [51]. We also included *B. albiflora*, a perennial herbaceous species from the subfamily Rutoideae. The divergence between *Boenninghausenia* and *Citrus* was estimated to have occurred in the Late Cretaceous, about 70 Mya, with an uncertainty range of 50–83 million years [52].

To further assess these divergence times, we extracted protein sequences for the selected species from the *Citrus* Pan‐genome to Breeding Database (http://citrus.hzau.edu.cn/download.php), as well as from a draft genome of *B. albiflora* (Z.J. Yang, unpublished). Using these protein sequences, we constructed a phylogenetic tree with MrBayes 3.2 [53]. The 13 Aurantioideae species showed an estimated divergence time of approximately 28.86 million years (Figure 1A). *B. albiflora* was inferred to have diverged from the Aurantioideae approximately 52 Mya, based on a fossil calibration using *Clausena* at 27.23 Mya [54]. These results were consistent with a phylogenetic tree generated with RaxML v8 [55] (Figure S1).

### 2.2 Development of Barcode FISH Probes for Chromosome Identification in *Citrus* Species

We employed the barcode‐FISH system [26] to identify every chromosome in a single FISH experiment. Two barcode probes, labeled in red and green, were designed based on the reference genome of *C. maxima* (http://citrus.hzau.edu.cn/index.php). The red probe contains 32598 oligos and the green probe 27737 oligos (45 nt each), targeting 14 and 12 distinct chromosomal regions, respectively, across the nine *C. maxima* chromosomes (Figure 1B). Each region is represented by an average of 2320 oligos (ranging from 2121 to 2465; Table S1). Together, the two probes are expected to produce 26 distinct FISH signals (Figure 1B), generating a unique color‐barcode pattern that enables unambiguous identification of all nine chromosomes in a single experiment.

### 2.3 Chromosome Identification in Three Foundational *Citrus* Species

**FIGURE 2:**
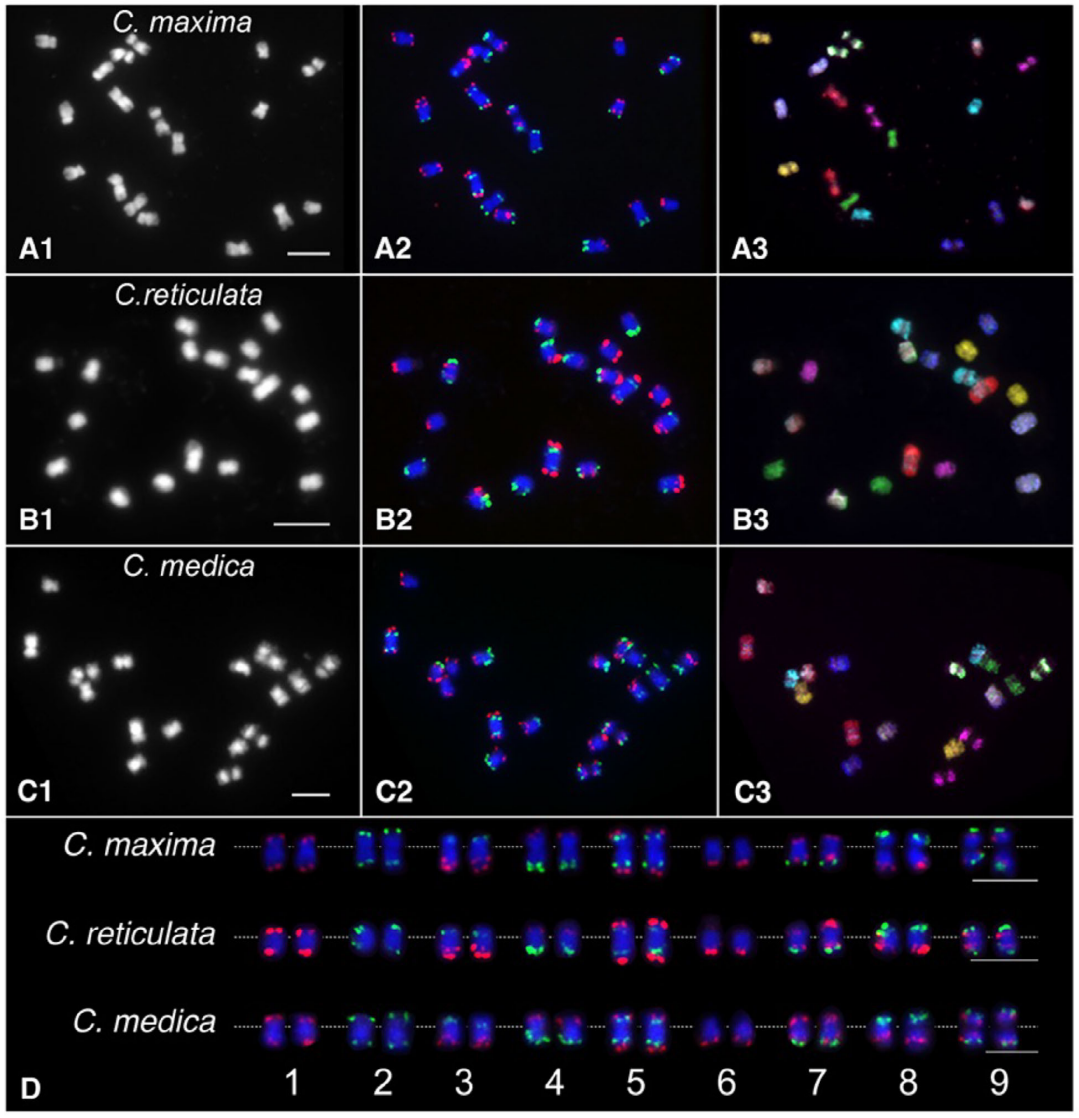
Barcode‐FISH‐based chromosome identification in three foundational *Citrus* species. (A1) A metaphase cell of *C. maxima* (monochrome). (A2) Barcode‐FISH mapping. (A3) Three successive rounds of chromosome‐specific painting, with each homologous pair shown in a distinct color. (B1) A metaphase cell of *C. reticulata*. (B2) Barcode‐FISH mapping of (B1). (B3) Chromosome painting of (B1). (C1) A metaphase cell of *C. medica*. (C2) Barcode‐FISH mapping of (C1). (C3) Chromosome painting of (C1). (D) Homologous chromosome pairs (1–9) digitally isolated from barcode‐FISH images in (A2), (B2), and (C2), with centromere positions aligned (dotted white line). Bars = 5 µm.

The two barcode FISH probes were first applied to metaphase chromosomes of *C. maxima*, *Citrus reticulata*, and *Citrus medica*—the three foundational species from which most cultivated *citrus* cultivars were developed [56, 57]. Metaphase chromosomes were prepared from root tips of the three species (Figure 2A1,B1,C1) and hybridized with the two barcode probes. The two probes produced punctate FISH signals in each species, yielding the expected 14 red and 12 green signals (Figure 2A2,B2,C2), which enabled unequivocal identification of the nine chromosome pairs (Figure 2D).

To further validate the chromosomal locations of the barcode‐FISH signals, the same metaphase cells were sequentially hybridized to a set of nine chromosome‐specific painting probes developed in *C. maxima* [43] (Figure 2A3,B3,C3). These results demonstrate that the individual chromosomes in the three species can be reliably identified with a single FISH experiment using the two barcode probes.

### 2.4 A Pachytene Chromosome‐Based Karyotype of *C. reticulata*

**FIGURE 3:**
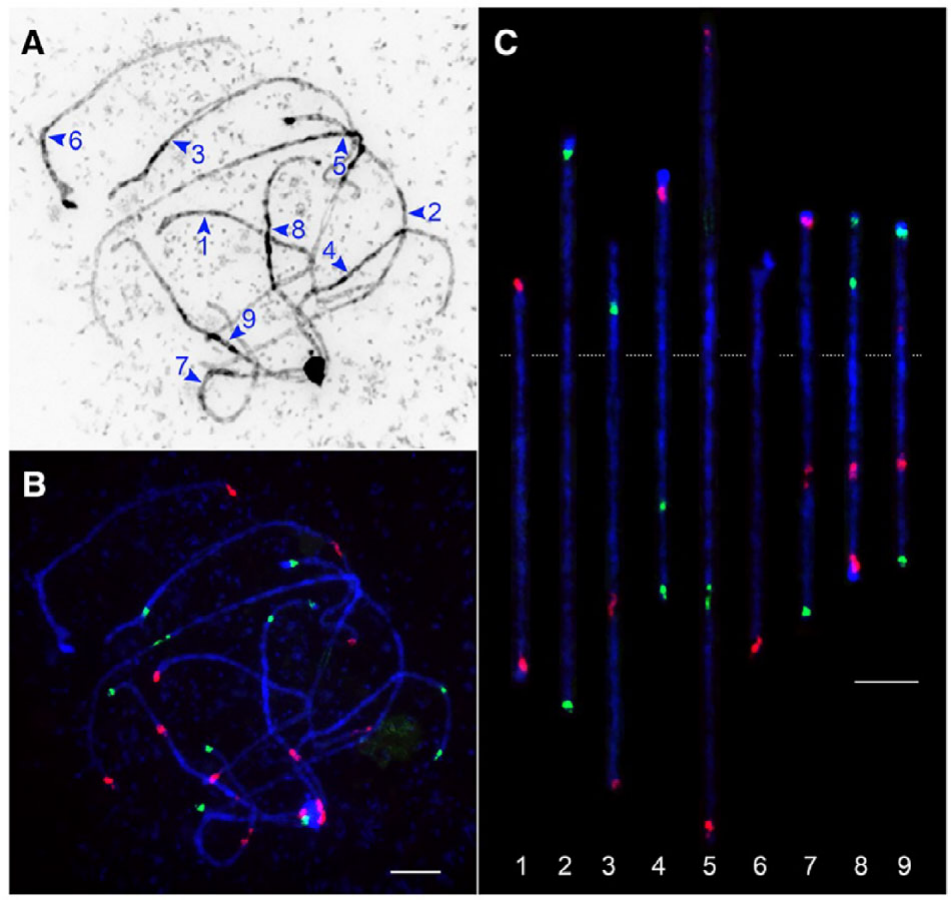
Identification of meiotic pachytene chromosomes in *C. reticulata*. (A) A DAPI‐stained pachytene cell is shown as a grayscale image to enhance contrast. Arrowheads indicate the positions of centromeres, which appear as lightly stained regions. Pachytene chromosomes 1 to 9 were identified by barcode‐FISH mapping in panel (B). (B) Barcode‐FISH mapping of the pachytene cell of (A). (C) Pachytene chromosomes 1 to 9 were digitally isolated from (B), straightened, and arranged from left to right. Centromere positions are aligned along a dotted line. Bars = 5 µm.

Meiotic pachytene chromosomes provide superior resolution for karyotyping compared to somatic metaphase chromosomes. However, a pachytene chromosome‐based karyotype has not yet been established in *Citrus* species, primarily due to the difficulty of unambiguously distinguishing individual pachytene chromosomes using traditional heterochromatin distribution patterns. To assess whether the barcode probes could facilitate pachytene chromosome identification, we prepared pachytene spreads from the *C. reticulata* cultivar “Red Mandarin” (Figure 3A). All 14 red and 12 green barcode signals were clearly detected across the nine pachytene chromosomes (Figure 3B), and their positions were consistent with the ideogram based on barcode distribution along the nine pseudomolecules (Figure 1B). In addition, terminal knobs that stained brightly with DAPI were observed on the short arms of chromosomes 2, 4, 6, 7, and 9, as well as on the long arms of chromosomes 1 and 8 (Figure 3C). These knobs were not visible on somatic metaphase chromosomes (Figure 2B1). Absolute chromosome lengths, relative lengths, and arm ratios were measured from ten pachytene cells, enabling the establishment of the first pachytene chromosome‐based karyotype in a *Citrus* species (Table 1).

**TABLE 1:** Chromosome lengths and arm ratios of *C. reticulata* pachytene chromosomes.

Chr.	Length (µm)	Relative length (%)^a^	Arm ratio^b^
1	35.80 ± 4.51	9.99 ± 0.73	3.77 ± 1.00
2	44.27 ± 5.52	12.37 ± 1.08	1.59 ± 0.28
3	45.70 ± 5.56	12.76 ± 0.96	2.79 ± 0.61
4	34.54 ± 4.50	9.67 ± 1.25	1.48 ± 0.29
5	63.28 ± 5.01	17.75 ± 1.50	1.46 ± 0.28
6	33.57 ± 5.88	9.39 ± 1.43	2.80 ± 0.40
7	34.39 ± 4.16	9.65 ± 1.22	1.87 ± 0.35
8	32.59 ± 5.27	9.11 ± 1.24	1.53 ± 0.26
9	33.48 ± 7.48	9.31 ± 1.62	1.49 ± 0.34

^a^ The length of an individual pachytene chromosome divided by the total complete length.

^b^ The length of the long arm divided by the short arm length of each pachytene chromosome.

### 2.5 Chromosome Synteny Among Distantly Related *Citrus* Species

**FIGURE 4:**
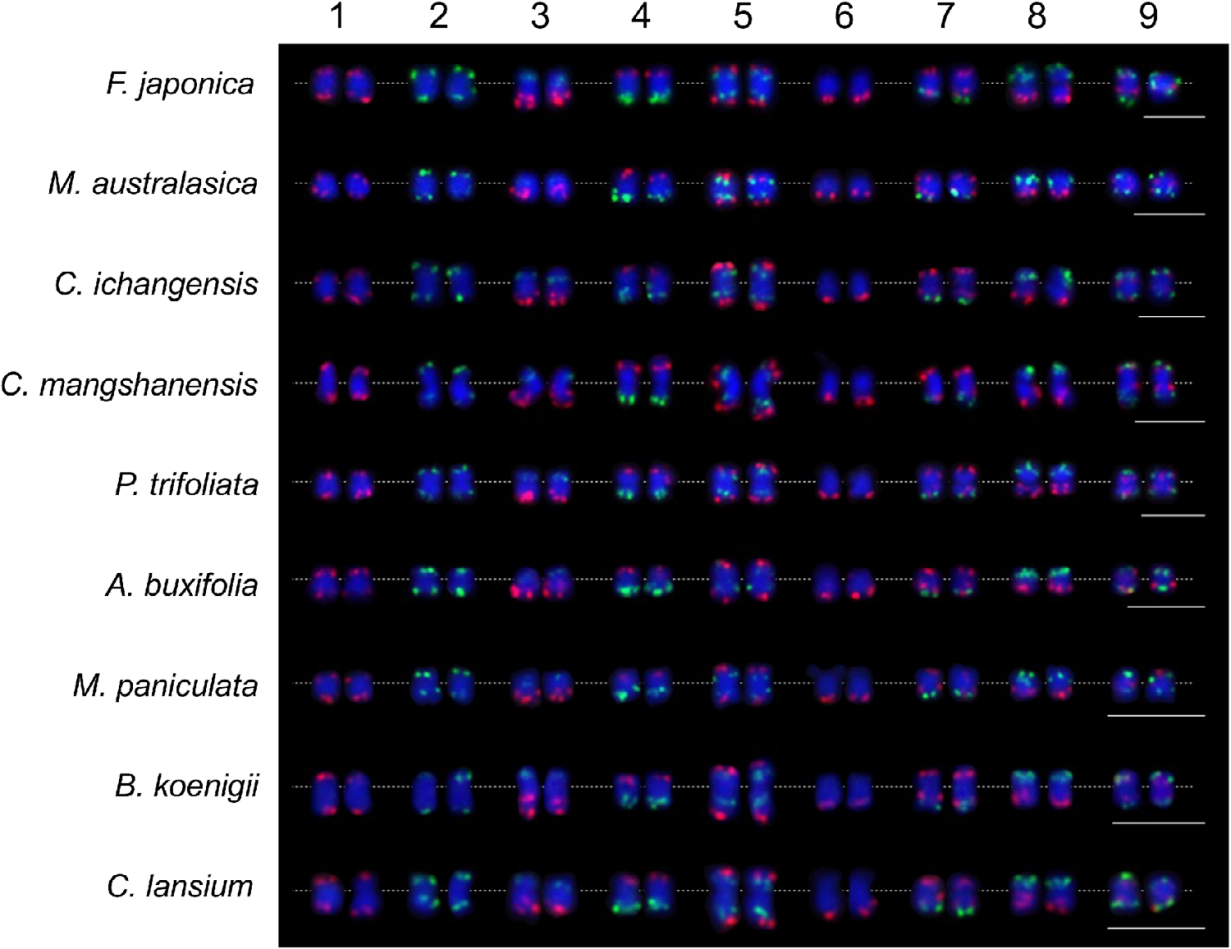
Comparative barcode‐FISH mapping and karyotyping of nine *Citrus*‐related species. The nine chromosome pairs from each species shown in Figure S2 were digitally isolated and arranged from left to right. For each species, the centromeric positions were aligned along a white dotted line. Identical barcode signal patterns were observed across all nine pairs of homologous chromosomes among the nine species. Bars = 5 µm.

To assess the utility of the barcode probes developed in *C. maxima*, we performed barcode‐FISH on nine representative species from the subfamily Aurantioideae. Several of these species, including *Bergera koenigii* and *Clausena lansium*, represent some of the most distantly related wild relatives of *Citrus* [58] (Figure 1A). Remarkably, the two probes produced strong and distinct hybridization signals on the chromosomes of all nine species (Figure S2). In addition, the barcode FISH signal patterns across the nine chromosomes were identical among all nine species examined (Figure 4). These findings indicate that the synteny across all nine chromosome pairs has been conserved despite nearly 29 million years of evolutionary divergence.

### 2.6 Complex Chromosome Rearrangements Associated with Sweet Orange Cultivar *Valencia*

Traditional sweet orange (*C. sinensis*) cultivars, developed prior to the modern breeding era, originated as hybrids between pummelo (*C. maxima*) and mandarin (*C. reticulata*) [59, 60]. Most modern sweet orange cultivars are derived from these traditional lines through bud mutations [56, 61]. *Valencia* is a widely cultivated sweet orange cultivar that arose as a bud mutant from a traditional cultivar. *Valencia* was known as a chromosomal translocation line based on its sterility, seedlessness, and meiotic chromosome behavior in pollen mother cells [62]. However, this proposed translocation was not confirmed by draft genome sequencing based on short‐read Illumina data [59]. More recently, a cytogenetic study using both oligo‐based and repetitive DNA probes identified a reciprocal translocation between chromosomes 4 and 9 in *Valencia* [63].

To further validate this translocation, we performed barcode‐FISH on *Valencia* chromosomes (Figure S3A2). Altered barcode signal patterns were observed in both copies of chromosome 4 and in one copy of chromosome 9 (Figure S3E). As a control, we analyzed another sweet orange cultivar, *Tarocco*, which exhibited standard barcode patterns across all nine chromosomes (Figure S3B2,E). Two additional hybrids, *Citrange* (a hybrid of *C. sinensis* × *P. trifoliata*) (Figure S3C2) and *C. aurantium* (a hybrid of *C. maxima* × *C. reticulata*) (Figure S3D2), also displayed normal barcode‐FISH patterns (Figure S3E). These results confirmed that the altered barcode patterns on chromosomes 4 and 9 are unique to *Valencia*.

**FIGURE 5:**
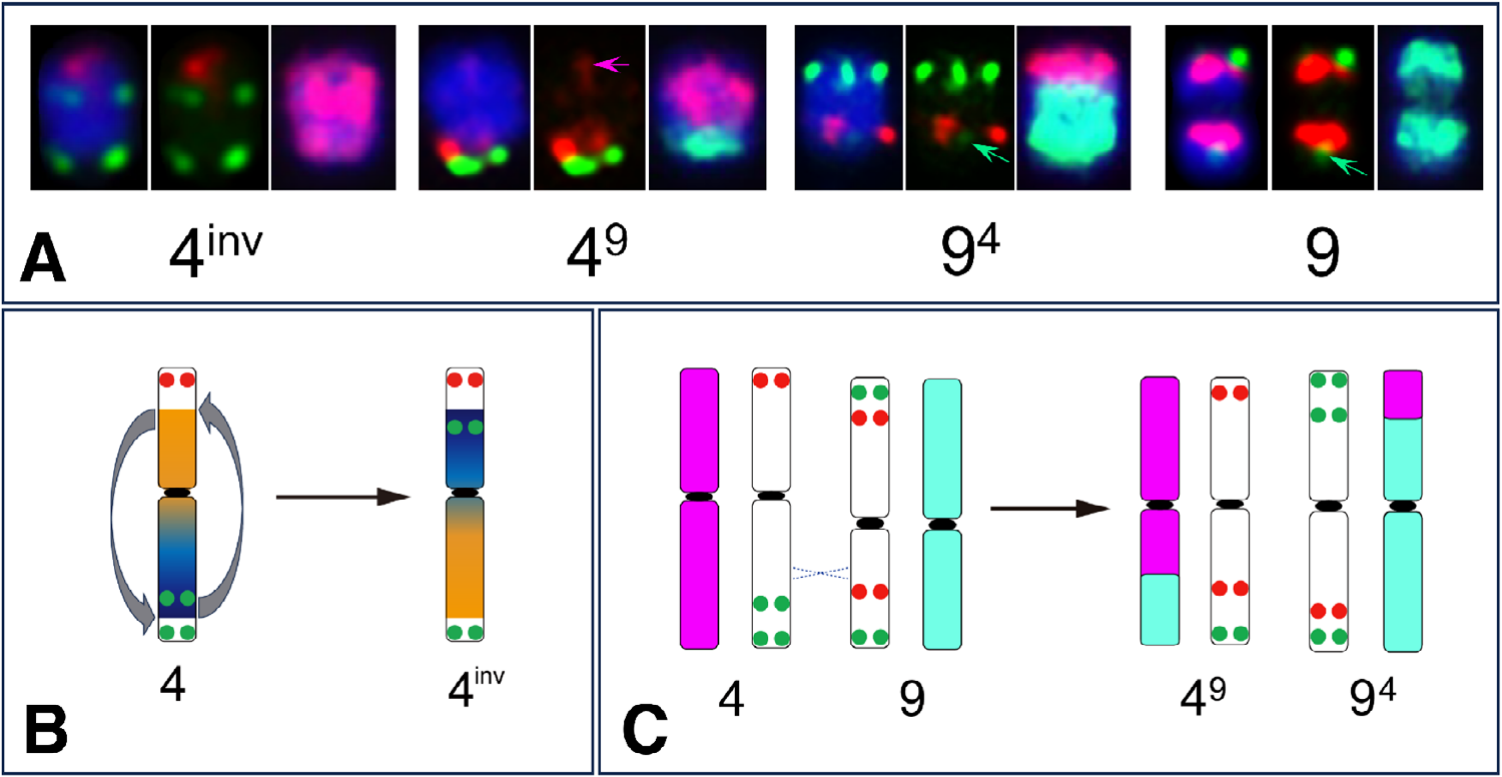
Characterization of chromosomal rearrangements in *C. sinensis* cv. *Valencia*. (A) Representative images of chromosomes 4^inv^, 4^9^, 9^4^, and 9, showing barcode‐FISH (left) and chromosome painting (right). The middle panels show barcode‐FISH signals extracted from the left images. Magenta and green arrows indicate relatively weak signals in these images. These individual chromosome images were isolated from the complete metaphase cell in Figure S4. (B) Schematic illustration of a pericentric inversion associated with one copy of chromosome 4. The inversion spans most of the chromosome and relocates a green barcode signal to the opposite arm. (C) Schematic illustration of a reciprocal translocation between chromosomes 4 and 9. The resulting translocation chromosomes, 4^9^ and 9^4^, exhibit distinct barcode signal patterns.

We next performed sequential barcode‐FISH and chromosome painting using painting probes specific to chromosomes 4 and 9, which confirmed the reciprocal translocation between these chromosomes (Figure S4). The altered barcode patterns further indicated that one copy of chromosome 4 in *Valencia* underwent a pericentric inversion, relocating a green signal from the long arm to the short arm, resulting in an inverted chromosome designated as 4^inv^ (Figure 5A,B). In addition, a reciprocal translocation between the second copy of chromosome 4 and one copy of chromosome 9 produced two translocated chromosomes, designated 4^9^ and 9^4^, respectively (Figure 5A,C). The sizes of the translocated segments were consistent with the observed alterations in barcode‐FISH signal patterns.

### 2.7 Characterization of Chromosomal Rearrangements in *Boenninghausenia albiflora*

**FIGURE 6:**
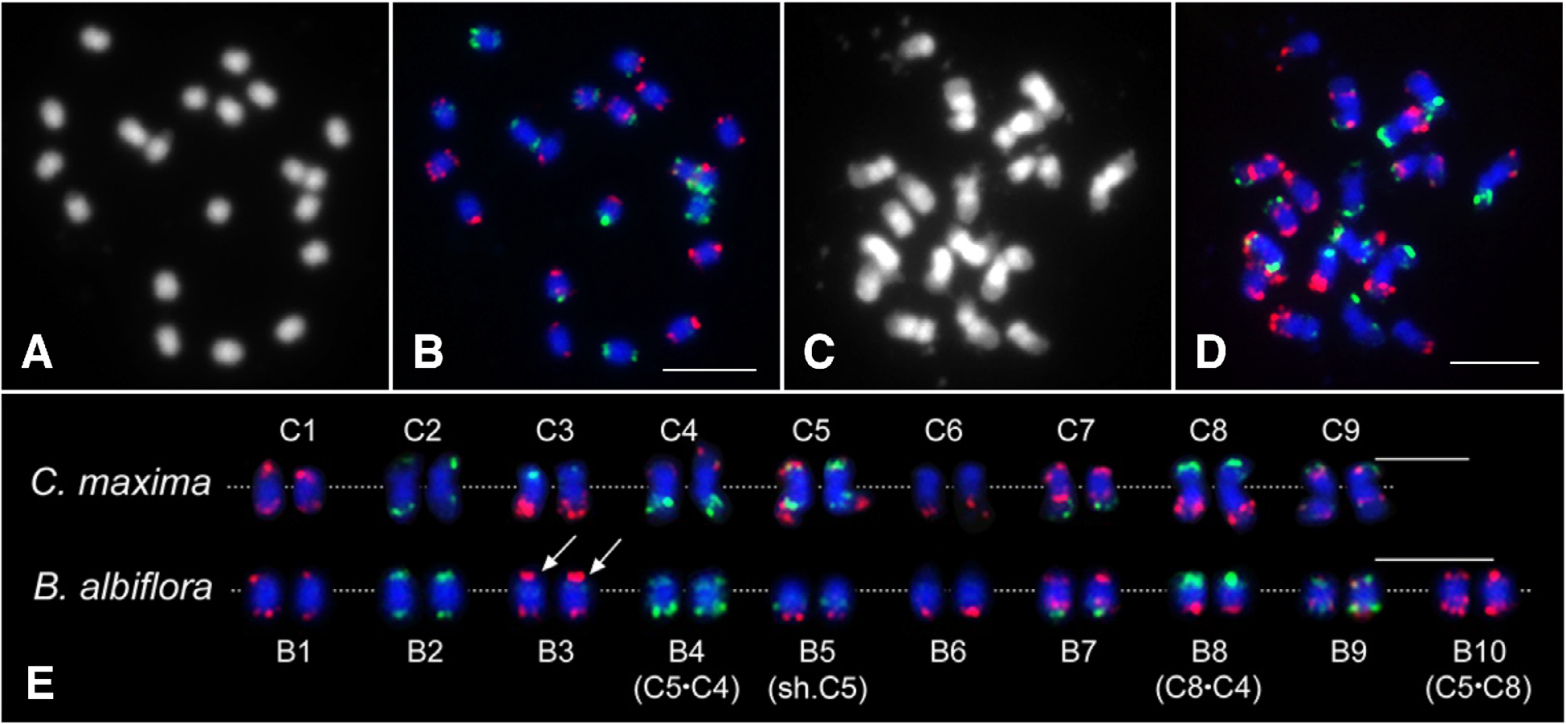
Comparative barcode‐FISH mapping between *C. maxima* and *B. albiflora*. (A) Chromosomes of a metaphase cell of *B. albiflora* are shown in monochrome. (B) Barcode‐FISH on the same metaphase cell. (C) Chromosomes of a metaphase cell of *C. maxima* are displayed in monochrome. (D) Barcode‐FISH of the same metaphase cell. (E) The *C. maxima* chromosomes (C1 to C9) and *B. albiflora* chromosomes (B1 to B10) were digitally extracted from (B,D), respectively. The alignment of the homoeologous chromosomes from the two species was based on similarity of the barcode signal patterns as well as chromosome painting information (Figure 7). The centromeres of the chromosomes in each species were aligned with dotted white lines. “sh. C5” indicates a truncated version of C5. Chromosomes C3 and B3 (arrows) displayed different barcode signal patterns, although both chromosomes hybridized exclusively to painting probe 3. Bars = 5 µm.

The base chromosome number for family Rutaceae is widely considered to be *n* = 9, although polyploids with *n* = 18, or multiples of 18, are also widespread within the family [64, 65, 66, 67, 68]. Interestingly, *B. albiflora* was previously reported to possess either 2n = 20 chromosomes [69, 70, 71], or 2n = 18 chromosomes [69, 71, 72, 73]. This discrepancy in chromosome number, potentially reflecting variation among different *B. albiflora* populations, prompted further investigation. In our study, we identified 20 chromosomes in an accession collected from the mountainous region near Chengdu, China (Figure 6A). Thus, this accession carries an additional chromosome pair relative to *Citrus* species.

**FIGURE 7:**
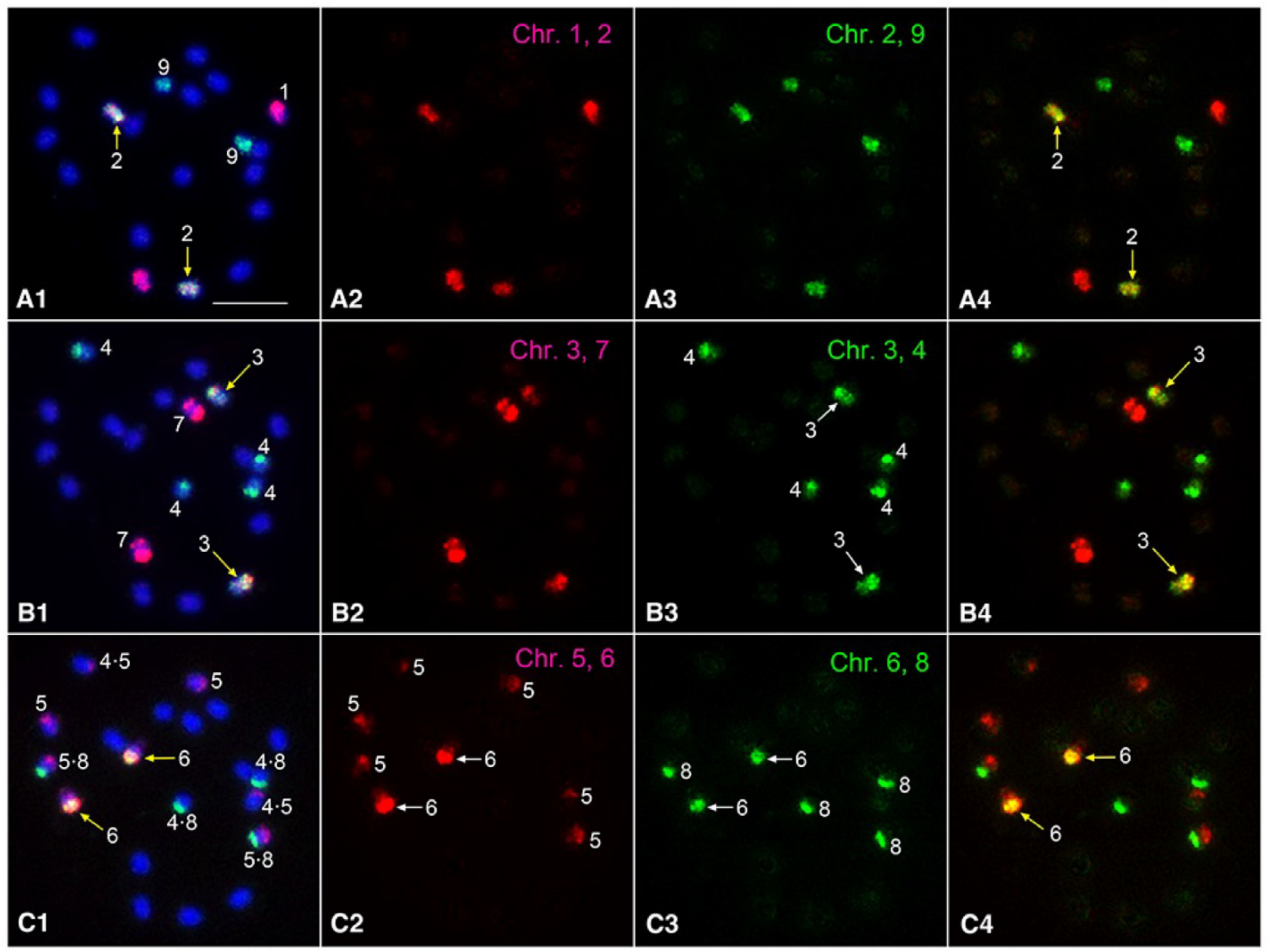
Characterization of chromosomal rearrangements in *B. albiflora* by sequential chromosome painting. The same metaphase cell from Figure 6B was sequentially re‐hybridized with painting probes developed from *C. maxima* chromosomes. (A1) Re‐hybridization with chromosome painting probes 1 (red), 9 (green), and 2 (yellow). Bar = 5 µm. (B1) Re‐hybridization with chromosome painting probes 7 (red), 4 (green), and 3 (yellow). **(C1)** Re‐hybridization with chromosome painting probes 5 (red), 8 (green), and 6 (yellow). Panels A2–A4, B2–B4, and C2–C4 show digitally extracted or merged signals from A1, B1, and C1, respectively. Specific hybridization signals are indicated by arrows and labels. The results reveal three distinct translocation chromosomes (4•5, 4•8, and 5•8), each hybridizing to two different painting probes (C1).

Remarkably, the two barcode probes produced strong, punctuated FISH signals on *B. albiflora* chromosomes (Figure 6B). The barcode patterns on five chromosome pairs closely resembled those of *C. maxima* chromosomes 1, 2, 6, 7, and 9 (Figure 6E), whereas the remaining chromosomes displayed modified patterns. We next attempted to delineate the evolutionary rearrangements of *B. albiflora* chromosomes by CCP using the nine painting probes developed in *C. maxima*. The same *B. albiflora* metaphase cell analyzed by barcode‐FISH (Figure 6B) was sequentially re‐hybridized in three rounds, each using three painting probes. Six probes hybridized exclusively to a single *B. albiflora* chromosome, corresponding to *C. maxima* chromosomes 1, 2, 9 (Figure 7A1), 3, 7 (Figure 7B1), and 6 (Figure 7C1). These results show that these six *B. albiflora* chromosomes have maintained the synteny with the corresponding *C. maxima* chromosomes.

In contrast, the *C. maxima* painting probe for chromosome 4 hybridized to two pairs of *B. albiflora* chromosomes (Figure 7B3). Similarly, probe 8 hybridized to two chromosome pairs (Figure 7C3), whereas probe 5 hybridized to three pairs (Figure 7C2). Integration of these painting patterns (Figure 7B1,C1) revealed three translocation chromosomes in *B. albiflora*: one composed of segments from *C. maxima* chromosomes 4 and 5 (C4•C5), a second from chromosomes 4 and 8 (C4•C8), and a third from chromosomes 5 and 8 (C5•C8) (Figure 7C1). In addition, one *B. albiflora* chromosome hybridized exclusively with painting probe 5 (Figure 7C1).

**FIGURE 8:**
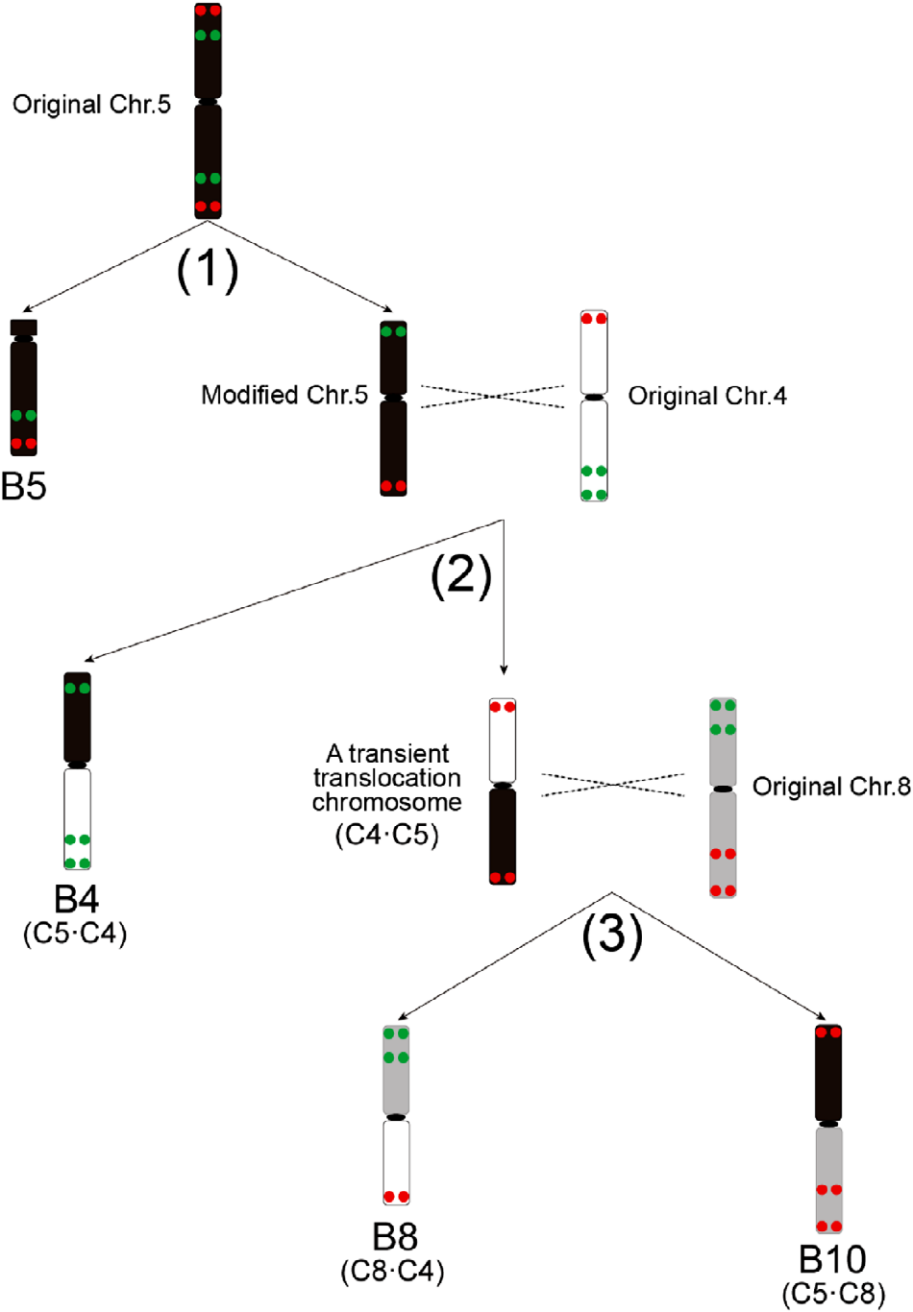
A model illustrating chromosome evolution in *B. albiflora*. (1) The original chromosome 5 (similar to C5) underwent multiple rearrangements, producing a truncated chromosome (B5) and another modified chromosome 5, characterized by the loss of a red signal on the short arm and a green signal on the long arm. These rearrangements altered the basic chromosome number from 9 to 10. (2) A reciprocal translocation occurred between the modified chromosome 5 and the original chromosome 4, generating chromosome B4 and a transient translocation chromosome. (3) A second translocation occurred between the transient translocation chromosome and original chromosome 8, resulting in chromosomes B8 and B10. Note: the breakpoints of the two proposed translocations are likely located near the centromeres of the chromosomes; however, their precise positions cannot be determined due to the limited resolution of chromosome painting.

Based on the combined barcode‐FISH and CCP results, we propose a model for chromosomal evolution in *B. albiflora*. The ancestral karyotype of *B. albiflora* likely consisted of 2n = 18 chromosomes, identical to that of the *citrus* species. Major rearrangements appear to have first involved chromosome 5, producing two derivatives: a truncated chromosome 5 (B5) and a structurally modified chromosome 5 (Figure 8). B5, which retained nearly only one arm of the ancestral chromosome, likely arose via a chromosome fission event. The modified chromosome 5 lost both the distal red signal on the short arm and the interstitial green signal on the long arm (Figure 8). These rearrangements involving chromosome 5 altered the basic chromosome number from 9 to 10. Subsequently, two sequential reciprocal translocations between the modified chromosome 5 and chromosomes 4 and 8 generated the present‐day configuration: chromosome B4 (C4•C5 translocation), chromosome B8 (C4•C8 translocation), and chromosome B10 (C5•C8 translocation) (Figure 8). Based on the positions of the primary constrictions on chromosomes B4, B8, and B10 (Figure 7B1,C1), the translocation breakpoints of all three chromosomes are located near the centromeres of their parental chromosomes (Figure 8). Collectively, the four *B. albiflora* chromosomes—B4, B5, B8, and B10—fit the current barcode signal patterns predicted by this model.

Despite the identification of four extensively rearranged chromosomes in *B. albiflora*, smaller‐scale rearrangements may remain undetected by barcode‐FISH and chromosome painting. While chromosome painting is especially powerful to reveal inter‐chromosomal translocations, it can not resolve intra‐chromosomal rearrangements. In addition, the resolution of barcode‐FISH may not allow characterization of small‐scale intra‐chromosomal rearrangements. For example, chromosome B3 of *B. albiflora* hybridized exclusively with the *C. maxima* painting probe 3 (Figure 7B1), yet chromosomes B3 and C3 display distinct barcode patterns (Figure 6E). This discrepancy could be explained by a large pericentric inversion; however, we cannot exclude the possibility that B3 also carries a small translocation with another chromosome. In this scenario, the exchanged segment may have been too small to be detected by the *C. maxima* painting probes.

## 3 Discussion

CCP was developed in mammalian species prior to the genome sequencing era and proved transformative for revealing synteny and chromosomal evolution across diverse mammalian species [5, 6, 13]. In plants, however, chromosome painting was not broadly feasible until the advent of oligo‐based FISH probes [25]. Since then, oligo‐based barcode‐FISH and chromosome painting have emerged as powerful tools in chromosome biology and as complementary approaches to DNA sequencing for genome analysis [24]. In this study, we generated barcode‐FISH and chromosome painting data for all chromosomes in *B. albiflora* within only a few weeks. Remarkably, these results were obtained from a single metaphase cell by sequentially re‐probing the same chromosome preparation (Figures 6B and 7A1,B1,C1), highlighting the efficiency of FISH‐based chromosome and genome mapping. Furthermore, once the karyotype of a species is established, the same FISH probes can be readily applied to additional accessions to assess population‐level variation in chromosome structure.

The success of comparative FISH experiments depends on the extent of sequence homology between the probe sequence and target DNA in cross‐species hybridization. For barcode probes, each signal is generated from 1000–2000 oligos designed from chromosomal regions spanning several hundred kb or a few Mb. If the corresponding regions in the target species have undergone rearrangements or extensive transposable element (TE) accumulation, the resulting barcode signals may be weakened or appear diffuse. For example, barcode probes developed in maize produced only a few distinct signals on sorghum chromosomes [27], largely because the maize and sorghum genomes have experienced substantial divergence through genome duplications and TE amplification, despite diverging only ∼12 Mya [74]. In contrast, potato (*Solanum tuberosum*) and tomato (*Solanum lycopersicum*), which diverged 8–9 Mya [75, 76], display excellent cross‐hybridization: barcode probes developed in potato generated clear and robust signals on tomato chromosomes. However, signal quality declined markedly on eggplant (*Solanum melongena*) chromosomes [26]. Eggplant diverged from the potato‐tomato lineage ∼15.5 Mya [77] and has a substantially larger genome (1170 Mb) [78] compared to potato and tomato (∼800–850 Mb) [79, 80].

Chromosome painting probes, owing to their much larger number of oligos, generally perform better than barcode probes in cross‐species hybridization. Nevertheless, their effectiveness declines with increasing evolutionary divergence. BAC‐based painting probes developed in *A. thaliana* have been successfully applied in CCP analysis across a large number of Brassicaceae species that diverged up to ∼20 Mya [81, 82]. Oligo‐based painting probes developed in potato have also been successfully applied across various *Solaunm* species but produced weak or indistinct signals in pepper (*Capsicum annuum*), which diverged from the potato‐tomato lineage ∼19.6 Mya [77]. Similarly, oligo‐based painting probes developed in cucumber (*Cucumis sativus*) successfully labeled homeologous chromosomes in related *Cucumis* species that diverged up to 12 Mya but failed in more distantly elated taxa [25]. Collectively, these studies suggest that, in most plant systems examined to date, the practical limit of cross‐species chromosome painting is approximately 20 million years of divergence.

In the present study, we demonstrate that oligo‐based FISH probes developed in *C. maxima* can be successfully applied to *B. albiflora*, a species that diverged more than 50 Mya—well beyond the previously recognized limit for comparative FISH mapping in plants. Both barcode and painting probes produced clear, punctate signals on *B. albiflora* chromosomes. This unexpected success may reflect a slower rate of sequence evolution in the *B. albiflora* lineage within Rutaceae. Despite their deep divergence, *B. albiflora* and *C. maxima* chromosomes remain similar in size (Figure 6), suggesting that their genome sizes have remained relatively stable. The absence of large‐scale genomic changes likely preserved chromosome structure and probe compatibility. Furthermore, most Rutaceae species are woody perennials, while only a few, including *B. albiflora*, are herbaceous. Woody plants are recognized for their exceptional karyotype stability [42], likely due to slower rates of genome evolution compared with annual or herbaceous taxa [83]. Taken together, these findings suggest that comparative FISH mapping with oligo‐based probes may be particularly effective in woody lineages and other taxa characterized by reduced genomic turnovers.

## 4 Conclusion

In summary, our study demonstrates that comparative FISH mapping can be extended to plant species that diverged more than 50 Mya. This expanded timescale underscores the power of comparative FISH to illuminate chromosome evolution far beyond closely related taxa. Much as Zoo‐FISH transformed the study of mammalian chromosome evolution two decades ago, comparative oligo‐FISH now provides plant biologists with a powerful framework to investigate genome and chromosomal evolution across both recent and ancient lineages. Our findings establish a foundation for extending cytogenomic research beyond model plant species, enabling comparative karyotype studies across the vast diversity of the plant kingdom. Moreover, our results suggest that comparative FISH mapping may be especially effective in plant lineages with slower rates of mutations and genome turnovers, where greater sequence conservation preserves the probe compatibility across highly diverse species.

## 5 Experimental Section

### 5.1 Plant Materials

Sixteen species were used for cytogenetic study, including three foundational *Citrus* species: *C. maxima* (Burm.) Merr. var. Sha‐tian pummelo, *C. reticulata* (Blanco) var. Red Mandarin, and *C. medica* (L.). Nine representative species were selected from the subfamily Aurantioidae: *Fortunella japonica* (Thunb.) Swingle, *Microcitrus australasica* (F. Muell.) var. Swingle (Australian finger lime), *Citrus ichangensis* (Ichang papeda, Swingle), *Citrus mangshanensis* (S.W. He & G.F. Liu) var. Wild Mangshan Mandarin, *Poncirus trifoliata* (L.) Raf., *Atalantia buxifolia* (Poir.) Oliv., *Murraya paniculata* (L.) Jack., *Bergera koenigii* (L.), and *Clausena lansium* (Lour.) Skeels.

Additional materials included two sweet orange (*C. sinensis*) cultivars, “*Valencia*” and “*Tarocco*”, *Citrange* (a hybrid of *C. sinensis* × *P. trifoliata*), *Citrus aurantium* (a hybrid of *C. maxima* × *C. reticulata*), and the perennial herb *B. albiflora* (Hook) Reichle. The *B. albiflora* accession was collected from a mountainous region near Chengdu, Sichuan Province, China (103.398806° E, 30.641569° N). All plant materials were maintained in the Resource Gardens of the National *Citrus* Engineering Research Center (Chongqing, China) and the Sichuan Academy of Agricultural Sciences (Chengdu, China).

### 5.2 Construction of Phylogenetic Tree

Protein sequences from 13 Aurantioideae species were extracted from the *Citrus* Pan‐genome to Breeding Database (http://citrus.hzau.edu.cn/download.php), along with sequences from a draft genome of *B. albiflora* developed by Z. J. Yang (unpublished). Orthologous groups were identified using BLASTP (e‐value < 1e‐5), followed by clustering with OrthoMCL [84]. Single‐copy orthologs were extracted and aligned using MAFFT v7.313 (L‐INS‐i option) with 1000 iterations [85]. Alignments were manually inspected and trimmed using trimAl v1.2 [86] to remove gaps and ambiguous regions with a coverage value of less than 60%. Phylogenetic analyses were conducted using both Bayesian inference (MrBayes v3.2) [53] and maximum‐likelihood (ML) (RAxML v8) [55] methods. The fossil of Clausena [54] was used as the constraint to estimate the divergent time by MCMCTree from PAML 4 [87].

### 5.3 Preparation of Mitotic and Meiotic Chromosomes

Mitotic chromosomes were prepared from root tips of germinated seeds following the protocol described previously [43]. Metaphase chromosomes were accumulated by treating root tips with nitrous oxide at 160 psi for 2 h in a stainless‐steel chamber. The root tips were then digested in an enzyme solution containing 2% cellulase (Yakult Pharmaceutical, Tokyo, Japan) and 1% pectinase (Sigma, St. Louis, MO, USA) at 37°C for 130–150 min. Meiotic pachytene chromosomes were prepared from anthers of young flower buds of *C. reticulata*, as previously described [88]. Flower buds were fixed in ethanol:acetic acid (3:1, v/v). Anthers containing pollen mother cells (PMCs) at the pachytene stage were selected, washed with distilled water for 20 min, and digested in 3% cellulase and 2% pectolyase (Plant Media, USA) at 37°C for 5 h.

All mitotic and meiotic chromosome preparations were performed using the dropping method [89]. After enzymatic digestion, root tips and anthers were washed in distilled water for 5 min and dissected in 1.5 mL tubes using a fine needle. The resulting cell suspensions (root tip cells or PMCs) were washed with 100% ethanol and resuspended in 20–40 µL of 3:1 acetic acid:methanol solution. For chromosome spreading, 8 µL of suspension was dropped from a height of ∼5 cm onto a clean glass slide.

### 5.4 FISH Mapping on Mitotic and Meiotic Chromosomes

Barcode probes were designed using Chorus2 software [90] following previously described procedures [26]. Probes were labeled with digoxigenin or biotin as previously described [25, 26]. FISH was performed under standard hybridization and washing stringency conditions [91]. Each hybridization mixture contained 300 ng of labeled probes per slide. For sequential rounds of chromosome painting after Barcode‐FISH, probes were removed by washing slides in 0.1× SSC at 55°C for 30 min after coverslip removal. Slides were then dehydrated through an ethanol series (70%, 90%, and 100% for 5 min each), followed by denaturation of chromosomal DNA in 70% formamide at 70°C for 2 min prior to the next round of hybridization with different probes.

Chromosomes were counterstained with 15 µL of VectaShield antifade mounting medium (Vector Laboratories, Burlingame, CA, USA). FISH images were captured using a CCD camera (ORCA‐Flash4.0; Hamamatsu, Japan) mounted on a Zeiss Axio Scope A1 fluorescence microscope. Pachytene chromosomes were isolated and straightened using ImageJ software (http://rsb.info.nih.gov/ij), and chromosome lengths were measured using the same software. Final image contrast was adjusted using Adobe Photoshop 5.0.

## Author Contributions

L.H. conceived the research. L.H. and J.J. designed the experiments. L.H., H.Z., X.Z., Z.Y., G.Z., G.L. generated the data. L.H., X.Z., W.L., and J.J. analyzed the data. J.H. and B.G. provided resources. J.J. and L.H. wrote the manuscript.

## Conflicts of Interest

The authors declare no conflicts of interest.

## Supporting information




**Supporting File**: advs75575‐sup‐0001‐SuppMat.pdf

## Data Availability

Data sharing is not applicable to this article as no new data were created or analyzed in this study.
