# Supplementary material for: Comparative Oligo‐FISH Mapping Illuminates Chromosomal Evolution Among Rutaceae Species Diverged Over 50 Million Years
Source: Adv Sci (Weinh). 2026 May 7;13(41):e21629. doi: 10.1002/advs.202521629 (PMC13336086; doi:10.1002/advs.202521629)
Supplement: Supplementary file 1 — Supporting File: advs75575‐sup‐0001‐SuppMat.pdf [file ADVS-13-e21629-s001.pdf]

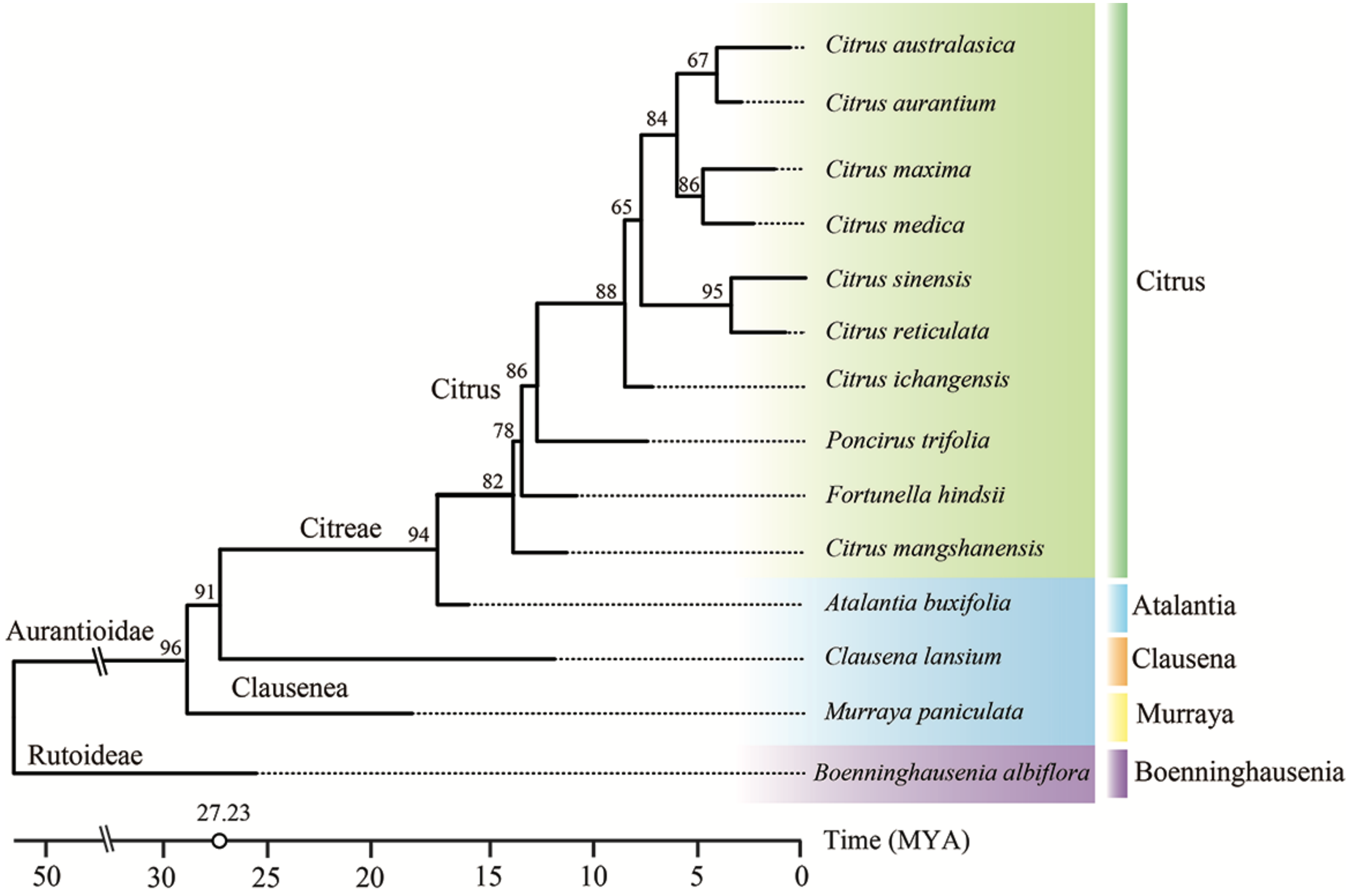

Figure S1. Phylogenetic tree of 14 species from the subfamilies Aurantioidae and Rutoideae. The tree was constructed by RAxML, consisting of one Rutoideae species (purple), three species from *Citrus*-related genera (blue), and ten species from *Citrus* genus (green).

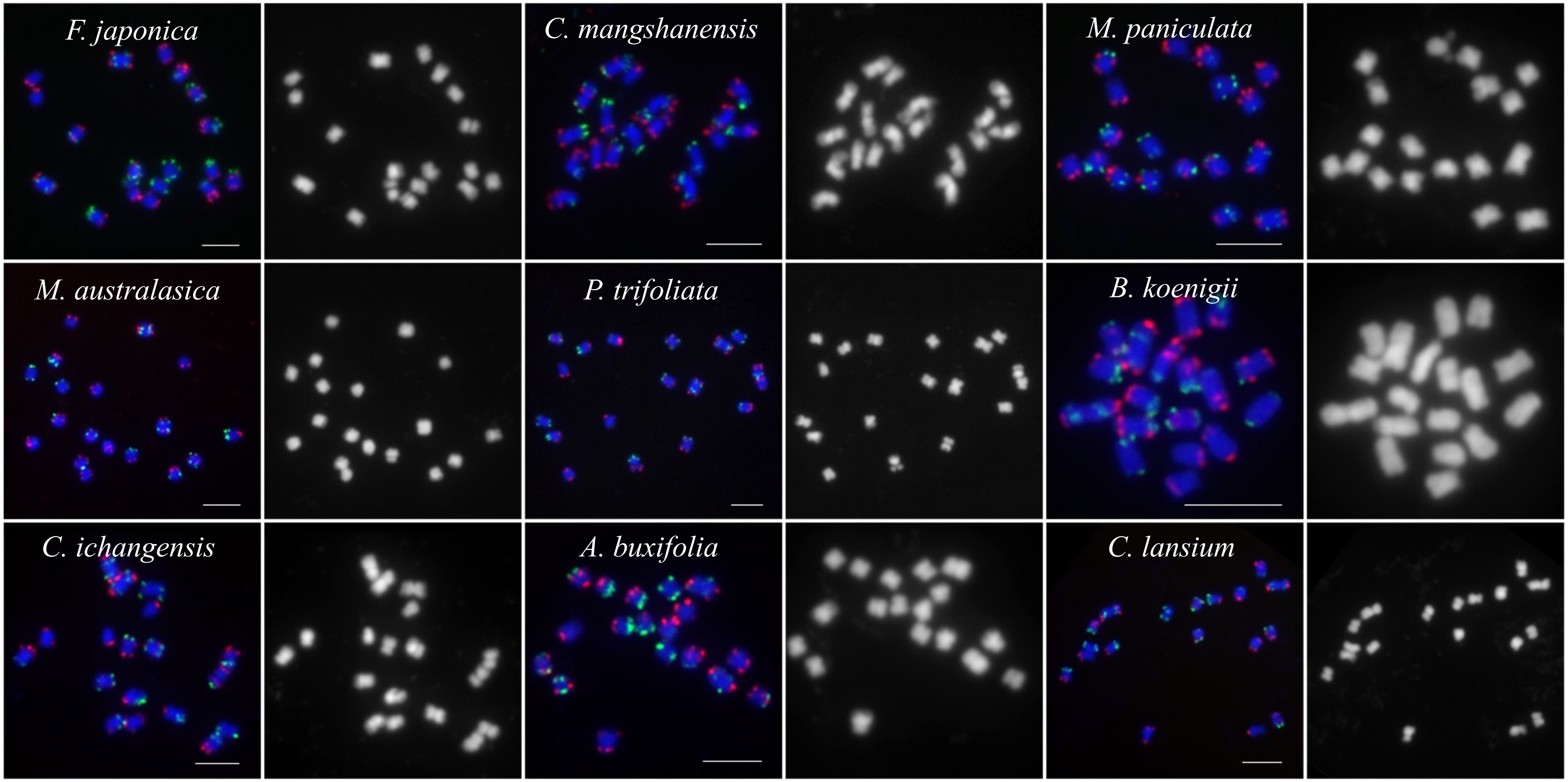

Figure S2. Barcode-FISH mapping on chromosomes of nine Aurantioidae species. For each species, the left panel shows the barcode-FISH, the right panel exhibits the chromosomes in the same metaphase cell in monochrome. Bars = 5 µm.

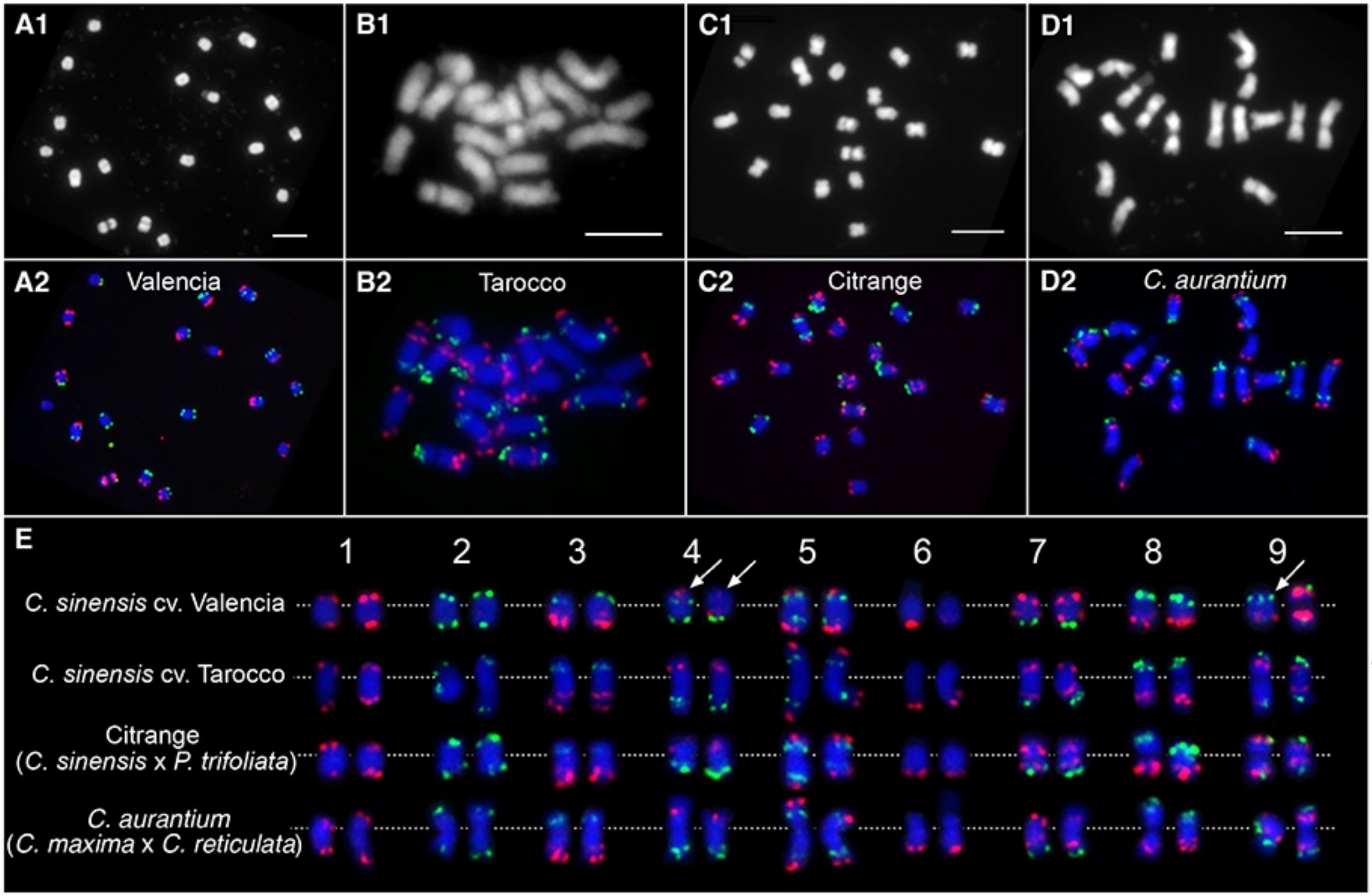

Figure S3. Barcode-FISH analysis of several *Citrus* hybrids. **(A1)** Metaphase chromosomes prepared from *C. sinensis* cv. *Valencia* shown in monochrome. **(A2)** Barcode-FISH on the chromosomes from (A1). **(B1)** Metaphase chromosomes prepared from *C. sinensis* cv. *Tarocco* in monochrome. **(B2)** Barcode-FISH on the chromosomes from (B1). **(C1)** Metaphase chromosomes prepared from Citrange in monochrome. **(C2)** Barcode-FISH on the chromosomes from (C1). **(D1)** Metaphase chromosomes prepared from *C. aurantium* in monochrome. **(D2)** Barcode-FISH on the chromosomes from (D1). **(E)** The nine pairs of homologous chromosomes were digitally isolated from (A2) to (D2). The centromeres are aligned with a dotted white line. Bars = 5 µm.

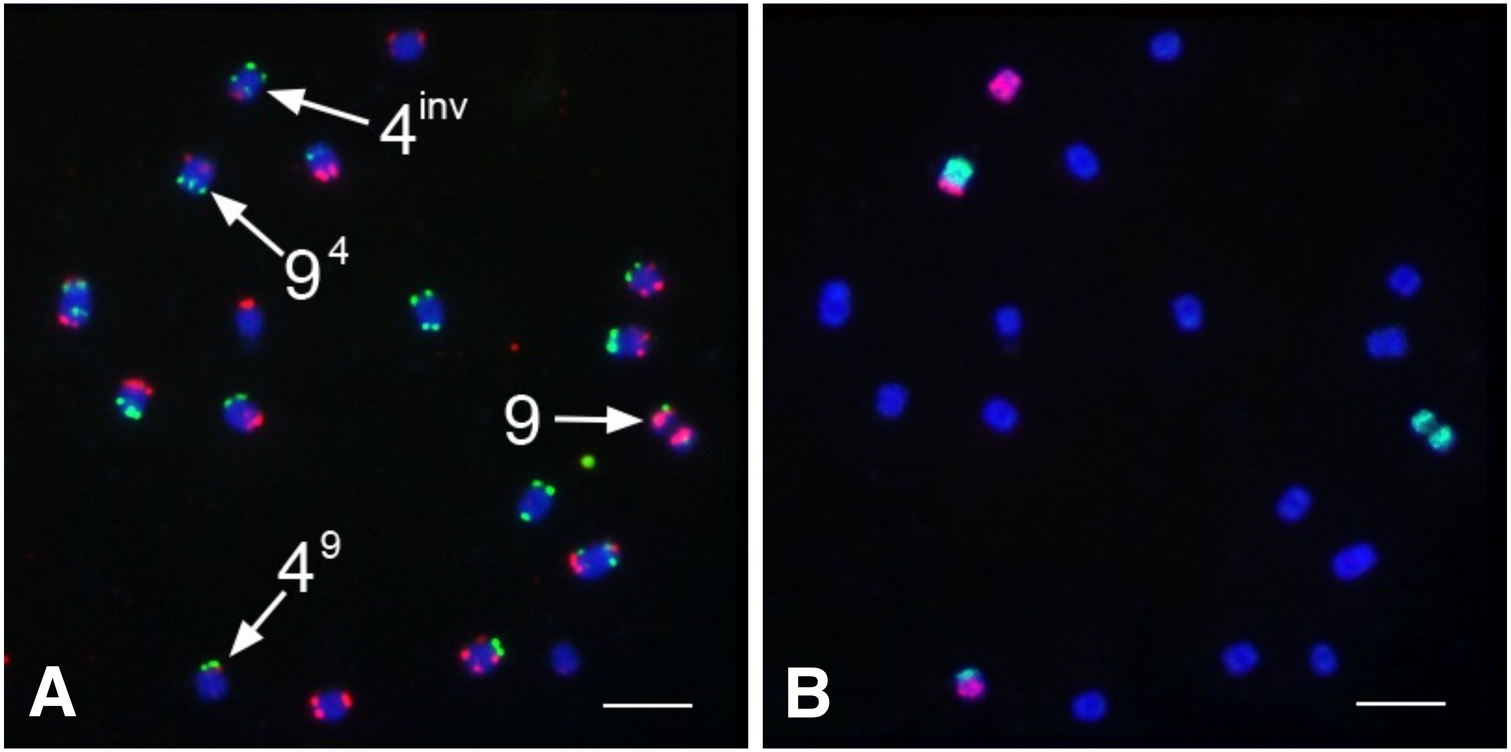

Figure S4. Barcode-FISH and chromosome painting in Valencia. **(A)** Barcode-FISH on a metaphase cell of *Valencia*. This is the same image shown in Figure S3A2. **(B)** The same metaphase cell was hybridized to painting probes for chromosome 4 (red) and chromosome 9 (green), respectively. Arrows indicate chromosome 9, inversion chromosome 4^{inv}, and translocation chromosomes 4^9 and 9^4. Bars = 5 µm.

Table S1. Oligo numbers and chromosomal positions of the 26 regions selected for the barcode probes

Chr.[a]	Arm	Oligo pool[b]	Start (kb)	End (kb)	Distance (Mb)[c]	Oligo number	Region length (bp)
Chr1.1	S	red	400	900		2424	500000
Chr1.2	L	red	31200	31900		2405	700000
Chr2.1	S	green	500	1000		2166	500000
Chr2.2	L	green	52050	52550		2352	500000
Chr3.1	S	green	2500	3320		2121	820000
Chr3.2	L	red	21950	22415		2219	465000
Chr3.3	L	red	30020	30500	7.605	2347	480000
Chr4.1	S	red	560	1090		2351	530000
Chr4.2	L	green	23200	23700		2339	500000
Chr4.3	L	green	28700	29200	5	2465	500000
Chr5.1	S	red	2	550		2287	548000
Chr5.2	S	green	8160	8700	7.61	2314	540000
Chr5.3	L	green	39600	40200		2404	600000
Chr5.4	L	red	48260	48750	8.06	2318	490000
Chr6.1	L	red	23020	23500		2422	480000
Chr7.1	S	red	50	600		2306	550000
Chr7.2	L	red	15550	16050		2293	500000
Chr7.3	L	green	21700	22150	5.65	2287	450000
Chr8.1	S	green	400	900		2346	500000
Chr8.2	S	green	3900	4600	3	2317	700000
Chr8.3	L	red	15750	16370		2349	620000
Chr8.4	L	red	20400	20900	4.03	2299	500000
Chr9.1	S	green	950	1400		2228	450000
Chr9.2	S	red	6600	7300	5.2	2267	700000
Chr9.3	L	red	31580	32300		2311	720000
Chr9.4	L	green	39500	40000	7.2	2398	500000

[a] Regions on each chromosome, e.g. Chr1.1: region 1 on chromosome 1.

[b] Oligo pool: red and green represent the two oligo pools that were synthesized, respectively.

[c] Distance: the distance between the two regions on individual chromsome arm.
